# Phylogeny of the Synlestidae (Odonata: Zygoptera), with an emphasis on *Chlorolestes* Selys and *Ecchlorolestes* Barnard

**DOI:** 10.1038/s41598-020-72001-x

**Published:** 2020-09-15

**Authors:** John P. Simaika, Jessica L. Ware, Rosser W. Garrison, Michael J. Samways

**Affiliations:** 1 IHE Delft Institute for Water Education, Westvest 7, 2611 AX Delft, The Netherlands; 2 Department of Conservation Ecology and Entomology, Stellenbosch University, P Bag X1, Matieland, 7602 South Africa; 3 Division of Invertebrate Zoology, American Museum of Natural History, New York, NY 10024 USA; 4 California Department of Food and Agriculture, 3294 Meadowview Road, Sacramento, CA 95832 USA

**Keywords:** Evolution, Phylogenetics

## Abstract

The Synlestidae (Odonata: Zygoptera) of southern Africa comprise some highly localized species. All but one species are endemic to South Africa, and many to the Cape Floristic Region. Here we present the first phylogenetic reconstruction of the southern African Synlestidae using nuclear and mitochondrial molecular data. The genera *Ecchlorolestes* and *Chlorolestes* are monophyletic, and we propose that the Neotropical family Perilestidae consisting of two genera, *Perilestes* and *Perissolestes,* be sunk within Synlestidae. We discuss the intra-familial relationships for the southern African Synlestidae.

## Introduction

### Distribution

Tillyard^1^ classified species of the family Lestidae as belonging to one of three subfamilies: the Epiophlebiinae, Lestinae, and Synlestinae. Subsequent to this classification, Tillyard^2^ then elevated the status of the subfamily Synlestinae to family level, as Synlestidae. Today, 33 species in the Synlestidae, commonly known as Malachites, are known from Africa, Australia, China, and the Island of Hispanola in the Caribbean. Seven species of *Chlorolestes* Sélys, 1862 (*C. apricans* Wilmot, 1975*; C. conspicuus* Hagen *in* Sélys, 1862*; C. draconicus* Balinsky*,* 1956; *C. elegans* Pinhey, 1950*; C. fasciatus* (Burmeister, 1839)*; C. tessellatus* (Burmeister, 1839); and *C. umbratus* Hagen *in* Sélys, 1862) and two species of *Ecchlorolestes* Barnard 1937 (*E. nylephtha* Barnard, 1937*; E. peringueyi* (Ris, 1921)) occur in southern Africa (see Fig. S1 for images live specimens and Fig. S2 for present-day species distributions), of which eight are endemic to South Africa, and one (*C. elegans*) to wider southern Africa^3^. Two Neotropical genera, *Perilestes* Hagen *in* Selys, 1862 and *Perissolestes* Kennedy, 1941, comprising Perilestidae, may be either sister to Synlestidae, or perhaps highly modified Synlestidae^4^. Southern African Synlestidae are the focus of this paper. Additional species included here are *Chromisagrion risi* Morton, *Episynlestes albicaudus* (Tillyard), *E. cristatus* Watson & Moulds*, E. intermedius* Theischinger & Watson***, Synlestes selysii*** Tillyard*, S. tropicus* Tillyard*,* and *S. weyersii* Selys, all of which are endemic to Australia. There are about 16 species in Asia (in two genera, *Sinolestes* Needham, 1930 and *Megalestes* Selys, 1868), of which six *Megalestes* species occur in India^5^. One species, *Phylolestes ethelae* Christiansen, occurs in the Caribbean^6^.

Here we present a comprehensive phylogenetic reconstruction of the southern African Synlestidae, using nuclear and mitochondrial molecular data. We then discuss the intrafamilial relationships for the southern African Synlestidae with respect to morphological synapomorphies, and comment on the current classification, suggesting that a revision of the group is needed.

## Methods

### Taxon sampling

All specimens were field collected. Of the seven known *Chlorolestes* species, we had specimens of each, as well as both species of *Ecchlorolestes*. Specimens used in the analyses here are: *C. apricans* (10 specimens), *C. conspicuus* (10 specimens), *C. elegans* (1 specimen), *C. fasciatus* (5 specimens), *C. tessellatus* (16 specimens), *C. umbratus* (10 specimens), *E. nylephtha* (6 specimens) and *E. peringueyi* (10 specimens). Despite several attempts, we were unable to amplify *C. draconicus*. We included all sequences of Synlestidae from NCBI (Table S2), which added an additional 14 28S and 8 COI sequences to our dataset.

### Gene selection, DNA extraction and PCR amplification

We amplified the nuclear 28S ribosomal DNA and the mitochondrial protein coding fragment cytochrome oxidase one (COI).These characters were included in the analysis and, where necessary, coded as missing for our taxa (in our analyses, some taxa had incomplete data; Table S1).

Muscle tissue was extracted using a Qiagen DNEasy tissue kit overnight at 55 °C with 180 µL of ATL Buffer and 20 µL Proteinase-K. Older specimens (collected prior to 1980) were extracted with 40 µL (twice the suggested amount) of Proteinase-K buffer for several days (2–5 days, depending on the age of specimen, with specimens older than 10 years old left for 2 days, older than 20 years left for 3 days, older than 25 years left for 4 days, and older than 30 years left for 5 days). All other steps followed the manufacturer’s protocol. PCR amplification was performed in 25-µl (total volume) mixtures by using Ready-To-Go PCR beads (GE Healthcare). We amplified the mitochondrial protein coding gene fragment COI, and the D2 and D3 regions of the 28S nuclear ribosomal subunit. PCR primers (D2ODup, D2DNB, D3up, D3dn) and their sources, are listed in Ware et al. (2007; but for COI we used the universal primers HCO and LCO from Folmer et al., 1994: LCO-1490 5′-GGT CAA CAA ATC ATA AAG ATA TTGG-3′, HCO-2198 5′-TAA ACT TCA GGG TGA CCA AAA AAT CA-3′). Programs used for amplifications were (a) 96 °C, 3 min; 94 °C, 30 s; 50 °C, 30 s; 72 °C, 45 s for 35–40 cycles; 72 °C, 10 min and (b) 96 °C, 3 min; 94 °C, 30 s; 46 °C, 30 s, 72 °C, 45 s for 10 cycles; 94 °C, 30 s; 48 °C, 40 s; 72 °C, 45 s for 30 cycles; 72 °C, 10 min. A Qiagen QIAquick PCR purification kit was used to purify amplified product (via silica-gel-membrane spin-column centrifugation, using buffers PB, PE and EB according to the manufacturer’s instructions), which was then sequenced on an ABI 3100 capillary sequencer. Sequences from forward and reverse strands were compared and edited in Sequence Navigator^7^.

### Alignment

Initial sequence alignments were made using Clustal-X^8^ and the resulting 28S files were then aligned manually in Microsoft Word using secondary structural models.

### Phylogenetic reconstruction

Data were partitioned and analyzed using IQTREE 2 (Trifinopoulos et al., 2016), and separate gene trees were also reconstructed. For the combined dataset, a GTR + F + R3 model^9^ was implemented; for the COI only tree a TIM2 + F + G4 model and for the 28S only tree a TN + F + R2 model were implemented.

#### Biogeographical analyses

We evaluated geographical patterns in Synlestidae using the parsimony ancestral state reconstruction function in Mesquite^10^. We assigned the taxa in our phylogeny to one or more of three biogeographical regions: Southern, New World, Central Africa and Australasia. We ran these analyses on our consensus maximum likelihood tree. We set no constraints, and taxa were considered to have equal ability to disperse to each area.

#### Morphology

We examined the morphological features of several adult Synlestidae (*Ecchlorolestes, Chlorolestes, Nubiolestes* Fraser, 1945) using standard stereo microscopy, to assess synapomorphies among and within the genera *Ecchlorolestes* and *Chlorolestes*. We describe these traits below. We evaluated trait evolution patterns using the parsimony ancestral state reconstruction function in Mesquite^10^. We ran these analyses on our consensus maximum likelihood tree.

**Figure 1:**
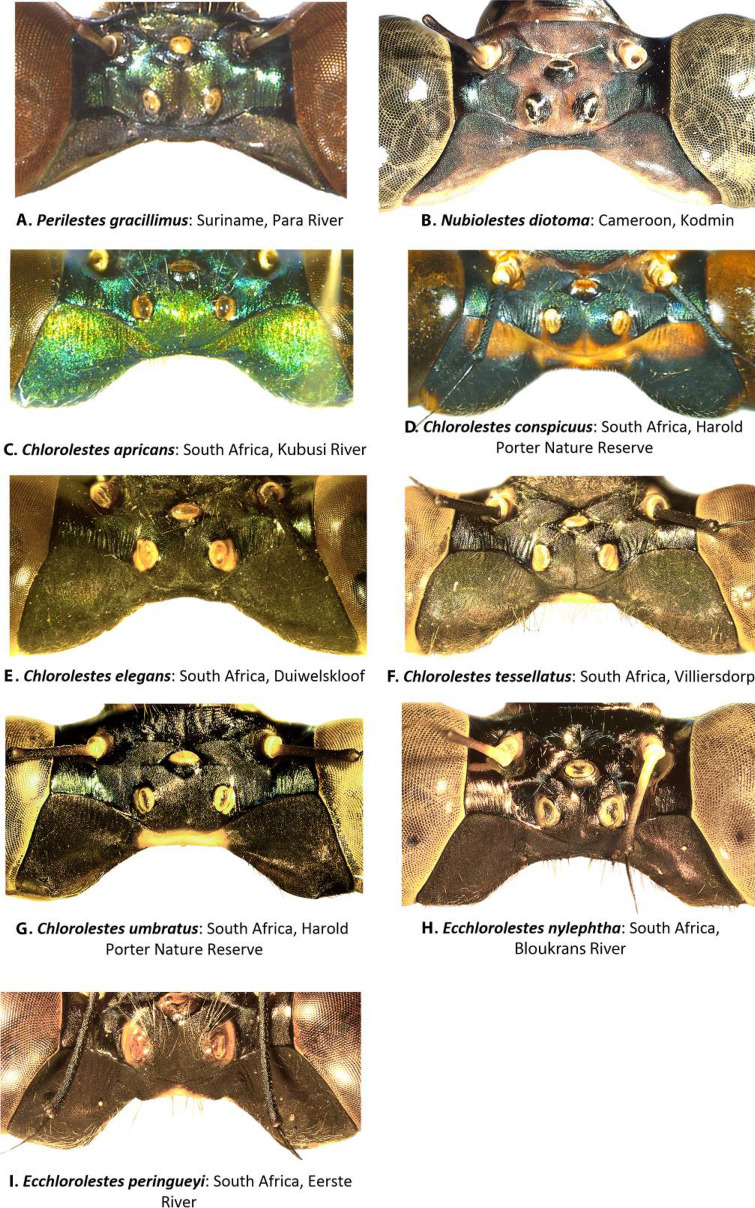
Female epicrania, dorsal view of: (**A**) *Perilestes gracillimus*, (**B**) *Nubiolestes diotoma*, (**C**) *Chlorolestes apricans*, (**D**) *C. conspicuus*, (**E**) *C. elegans*, (**F**) *C. tessellatus*, (**G**) *C. umbratus*, (**H**) *Ecchlorolestes nylephtha*, and (**I**) *E. peringueyi*.
- Wing venation (Fig. 2): In terms of odonate species traits, wing veins are often used to distinguish among taxa. RP_3_ originates at or just beyond the subnodus in all species of *Chlorolestes* (Fig. 2C) and in *Nubiolestes* (Fig. 2B) and one cell beyond in Perilestidae (Fig. 2A) and originates before the subnodus in both species of *Ecchlorolestes* (Fig. 2D).

- Head traits (Fig. 1): There was no discernible difference among *Ecchlorolestes* and *Chlorolestes* in their epicrania or labra; *Chlorolestes conspicuus* (Fig. 1D) and *Ecchlorolestes peringueyi* (Fig. 1I) and, to a lesser extent, *C. umbratus* (Fig. 1G) have large postocular lobes. Pronounced postocular lobes are absent in *Perilestes* (Fig. 1A) and *Nubiolestes* (Fig. 1B).

**Figure 2:**
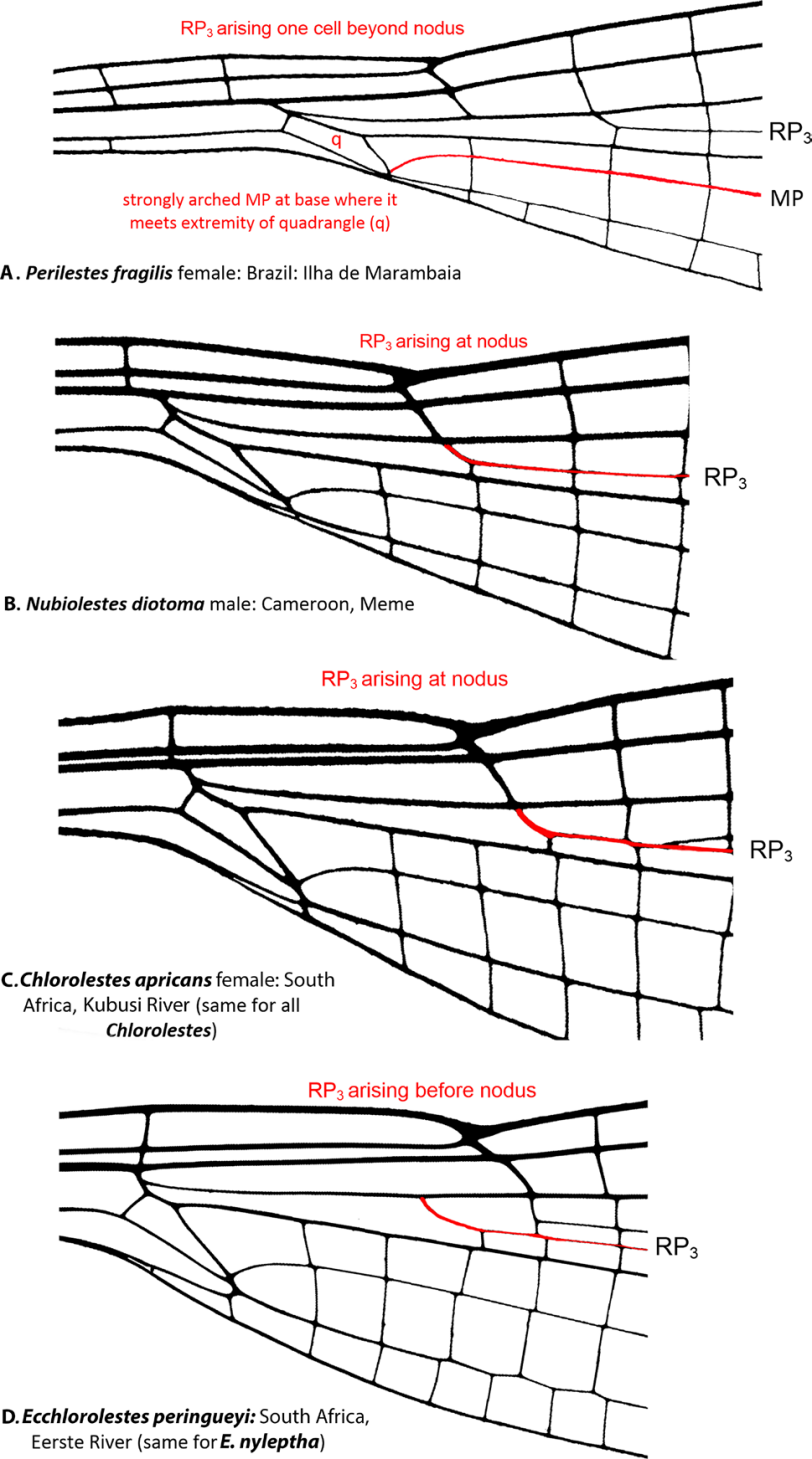
Basal wing venation details of: (**A**) *Perilestes fragilis* showing strongly arched MP at base of quadrangle (q), a synapomorphy for all genera included within Synlestidae, (**B**) *Nubiolestes diotoma*, (**C**) *Chlorolestes apricans* and (**D**) *Ecchlorolestes peringueyi* all showing origin of RP_3_ in relation to nodus.
- Genital ligulae (Fig. 3): Four types of genital ligula were observed among the Synlestidae examined here. In *Ecchlorolestes*, a sclerotized medial spine on the ligula is absent (Fig. 3E) as in *Perilestes* (Fig. 3A); in *Chlorolestes*, *C. apricans, C. conspicuus* and *C. umbratus* possess a scleritized medial spine shaped like a scimitar blade with a hollow canal/channel at its tip (Fig. 3C), perhaps for sperm transfer. *Chlorolestes draconicus*, *C. elegans, C. fasciatus* and *C. tessellatus* have a flexible medial spine on the genital ligula with a flap that covers the tip of the ligula, much like a pitcher plant (Fig. 3D). This is perhaps similar to the *Nubiolestes* form with a sclerotized medial spine tip in the form of a funnel on the genital ligula (Fig. 3B). Kennedy^11^ examined *C. fasciatus* and *C. tessellatus* and described a hood-like structure present covering the penis tip in the secondary genitalia of *Euchlorolestes* (synonymised as *Chlorolestes* by Barnard^12^). This flap-like structure is similar to that of *Nubiolestes* (Fig. 3B), whose ligula ends in a narrow tip, shaped like a partially flattened funnel.

**Figure 3:**
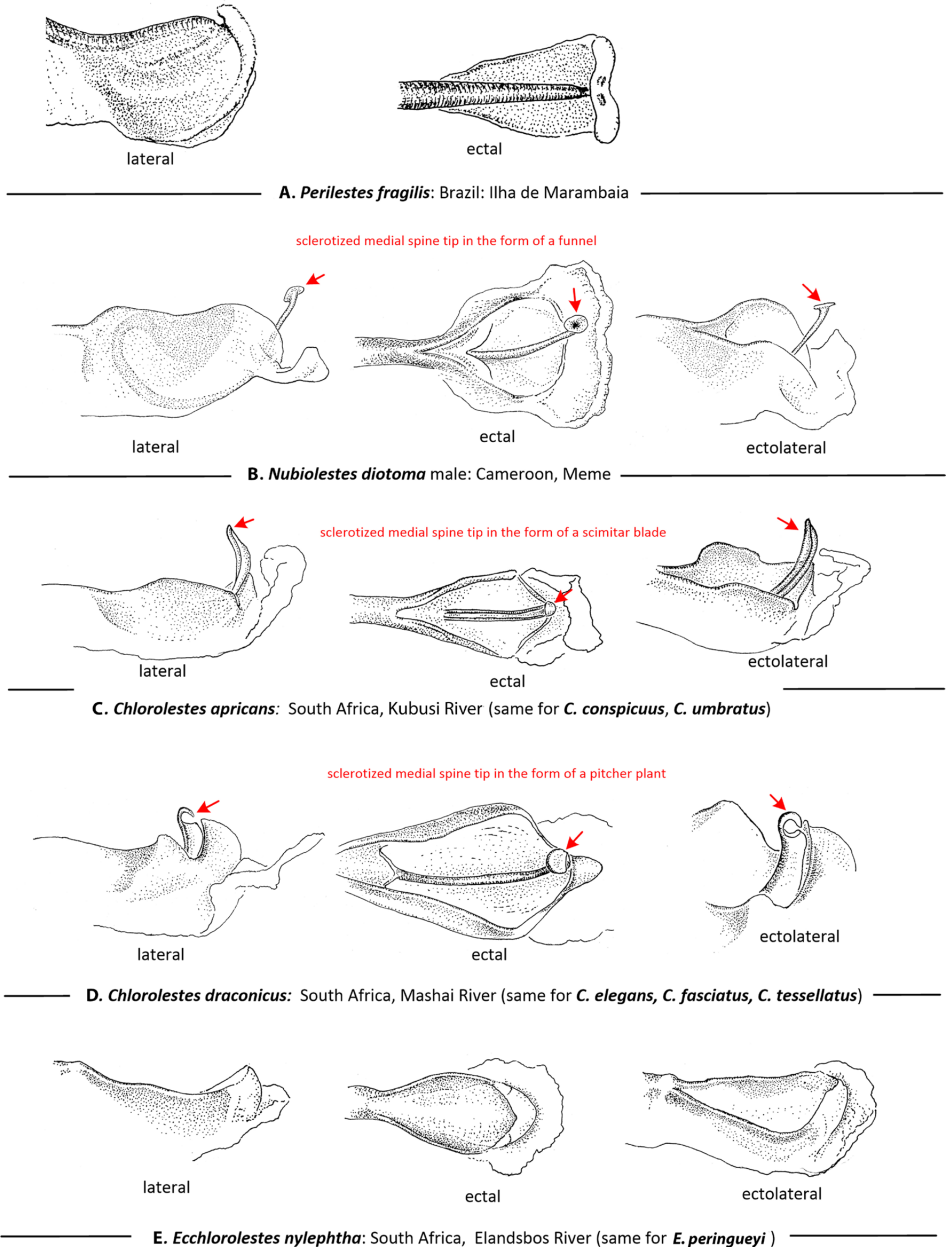
Lateral ectal and ectolateral views of apical segment of genital ligula of: (**A**) *Perilestes fragilis*, (**B**) *Nubiolestes diotoma*, (**C**) *Chlorolestes apricans*, (**D**) *Chlorolestes draconicus* and (**E**) *Ecchlorolestes peringueyi*. Heterospecific armature noted in red.
- Male caudal appendages (Fig. 4): Barnard^12^ evaluated the Synlestidae caudal appendages, which were subsequently treated by Pinhey^13^, Tarboton and Tarboton^14,15^ and Samways^16^, and are shown here in more detail. In *Chlorolestes* (Fig. 4D–J) and *Ecchlorolestes* (Fig. 4K,L), the paraproct varies in shape specifically. *Ecchlorolestes* is distinguished from *Chlorolestes* by the presence of a basal spine on the cercus. Two images of *Perilestes* (Fig. 4A,B) are illustrated here for comparison; they are most similar to *Nubiolestes* (Fig. 4C) in possessing an expanded distomedial lobe (absent in *Chlorolestes* and present only in *Ecclorolestes peringueyi*, Fig. 4L).

**Figure 4:**
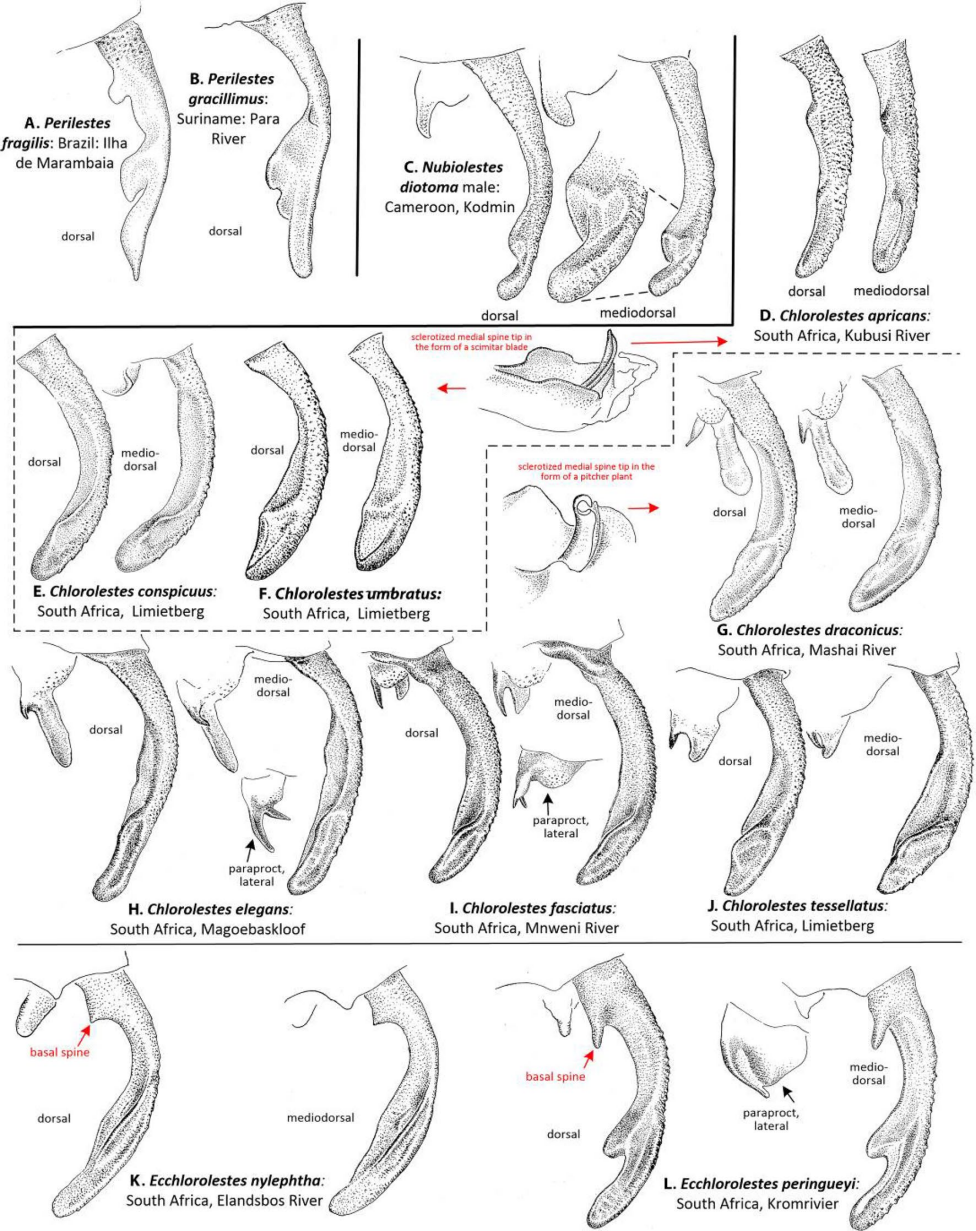
Dorsal and mediodorsal views of right cercus and paraprocts (the latter where shown in lateral view) of: (**A**) *Perilestes fragilis*, (**B**) *Perilestes gracillimus*, (**C**) *Nubiolestes diotoma*, (**D**) *Chlorolestes apricans*, (**E**) *C. conspicuus*, (**F**) *C. umbratus*, (**G**) *C. draconicus*, (**H**) *C. elegans,* (**I**) *C. fasciatus,* (**J**) *C. tessellatus*, (**K**) *Ecchlorolestes peringueyi* and (**L**) *E. peringueyi*. Appendages of *Chlorolestes apricans* (**D**), *C. conspicuus* (**E**), and *C. umbratus* (**F**) have genital armature in the form of a scimitar blade; appendages of *C. draconicus* (**G**), *C. elegans* (**H**)*, C. fasciatus* (**I**), and *C. tessellatus* (**J**) have genital armature in the form of a pitcher plant.
- Female ovipositor (Figs. 5 and 6): Two distinct types occur in *Ecchlorolestes* and *Chlorolestes*. The ovipositor teeth are large, robust and with an apically evenly convex blade in *Chlorolestes* (Fig. 5C–I), with each series of teeth separated by concavities (e.g. Fig. 5H). In *Ecchlorolestes*, ovipositor teeth are small, in a linear series approximate to one another (Fig. 6). *Chlorolestes tessellatus* (Fig. 5H) possesses an abbreviated ovipositor similar to *Nubiolestes* (Fig. 5B). The ovipositor of *Nubiolestes* is intermediate that of Synlestidae (Fig. 5C–I) and Perilestidae (Fig. 5A), perhaps providing support for Perilestidae being included within Synlestidae.

**Figure 5:**
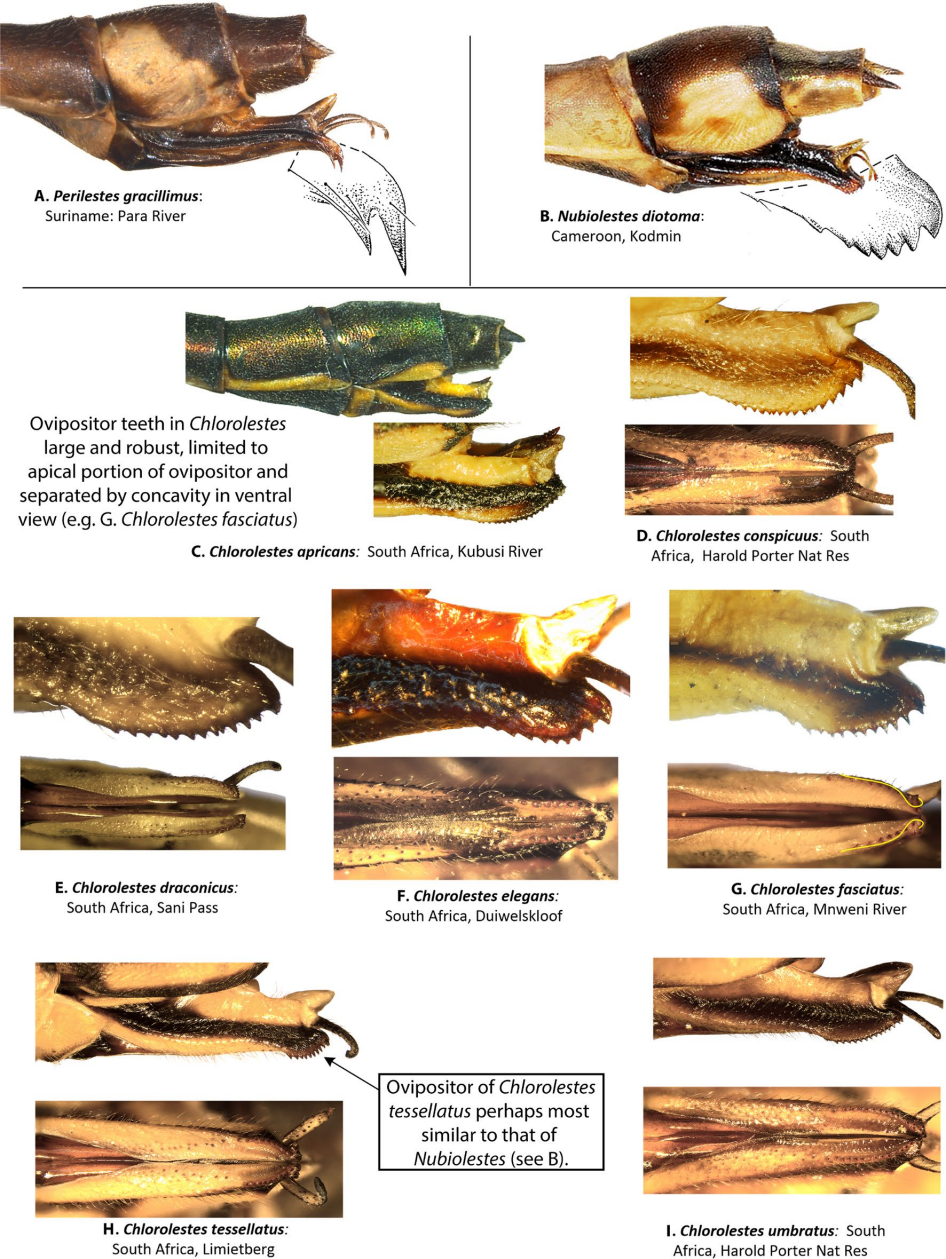
Lateral and ventral views of abdominal segments 9, 10 and ovipositor of: (**A**) *Perilestes gracillimus*, (**B**) *Nubiolestes diotoma*, (**C**) *Chlorolestes apricans*, (**D**) *C. conspicuus*, (**E**) *C. draconicus*, (**F**) *C. elegans*, (**G**) *C. fasciatus,* (**H**) *C. tessellatus,* and (**I**) *C. umbratus*.

**Figure 6:**
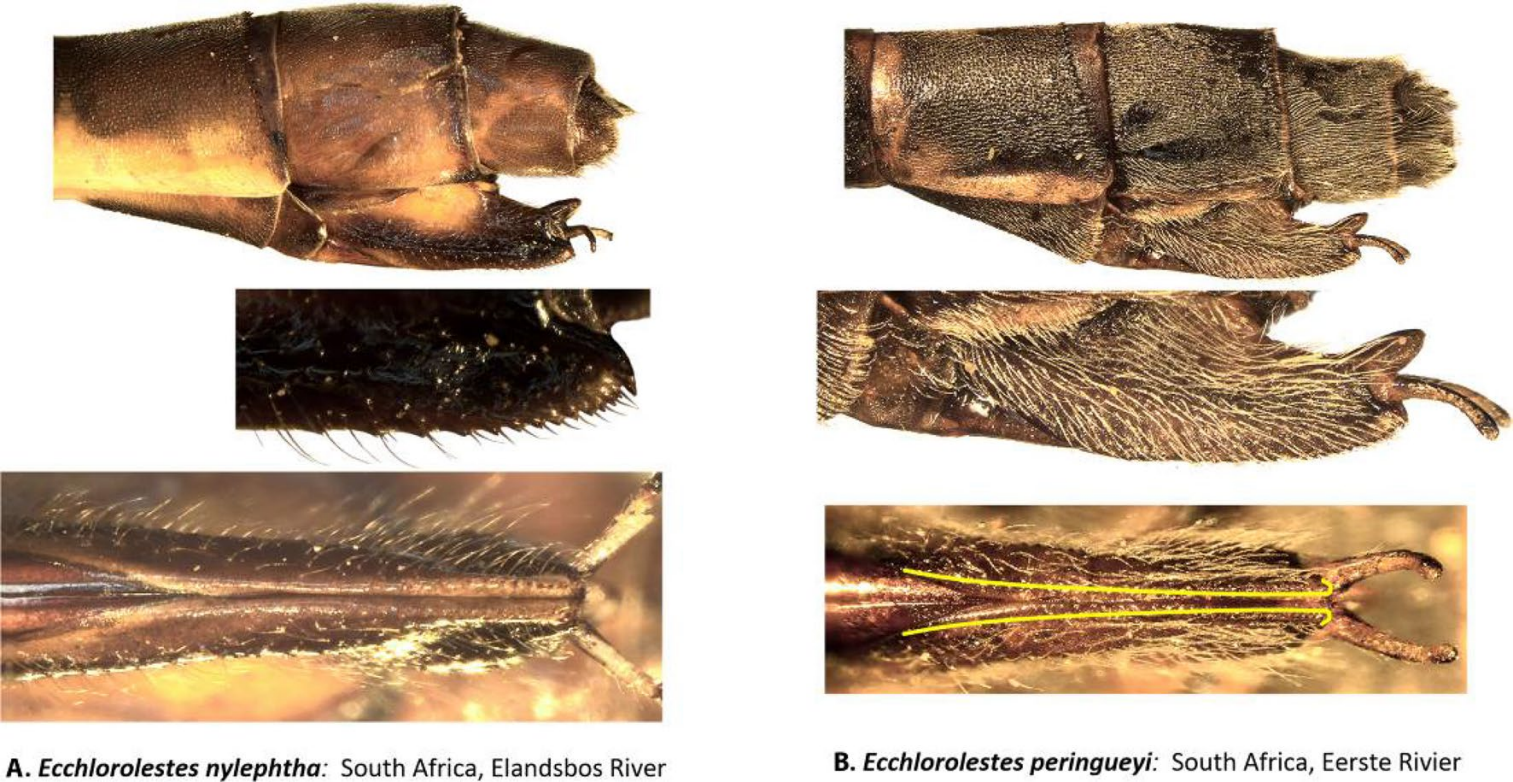
Lateral view of abdominal segments 9, 10 and ovipositor of: (**A**) *Ecchlorolestes nylephtha*, and (**B**) *E. peringueyi*. Note that the ovipositor teeth in *Ecchlorolestes* are small and in a linear series approximate to one another in ventral view.

## Results

**Figure 7:**
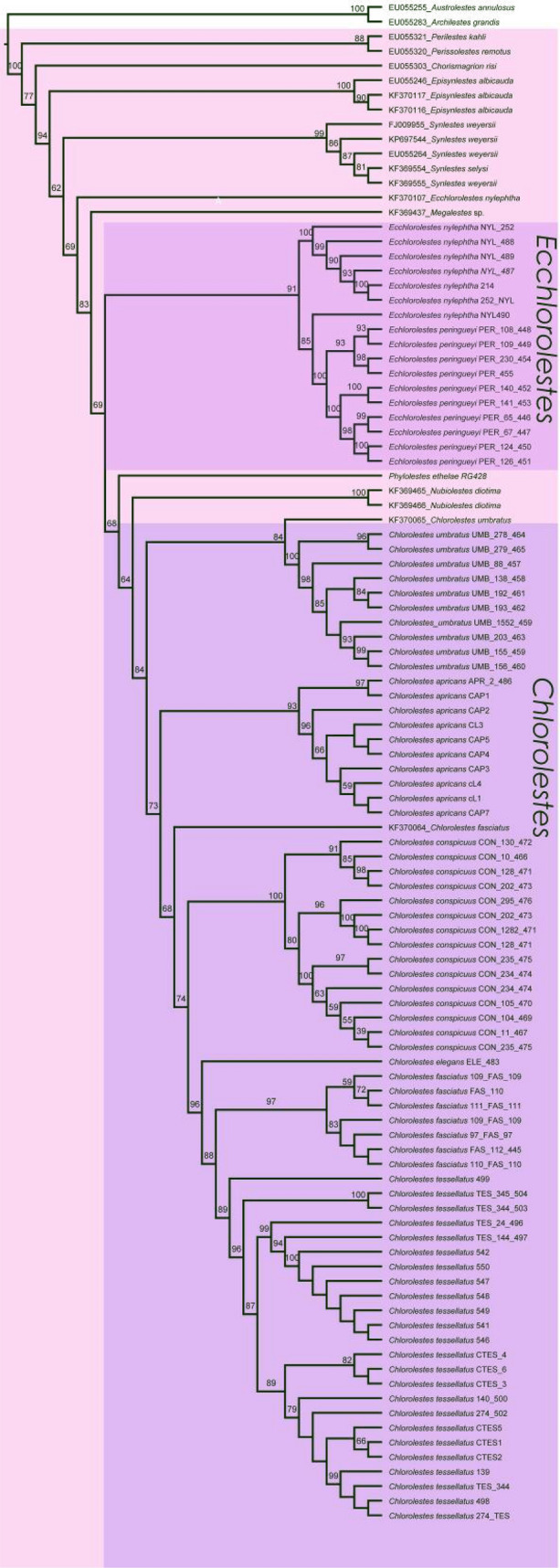
IQtree consensus tree, with morphological groups labelled (**A**–**D**) and bootstrap values reported above branches.

**Figure 8:**
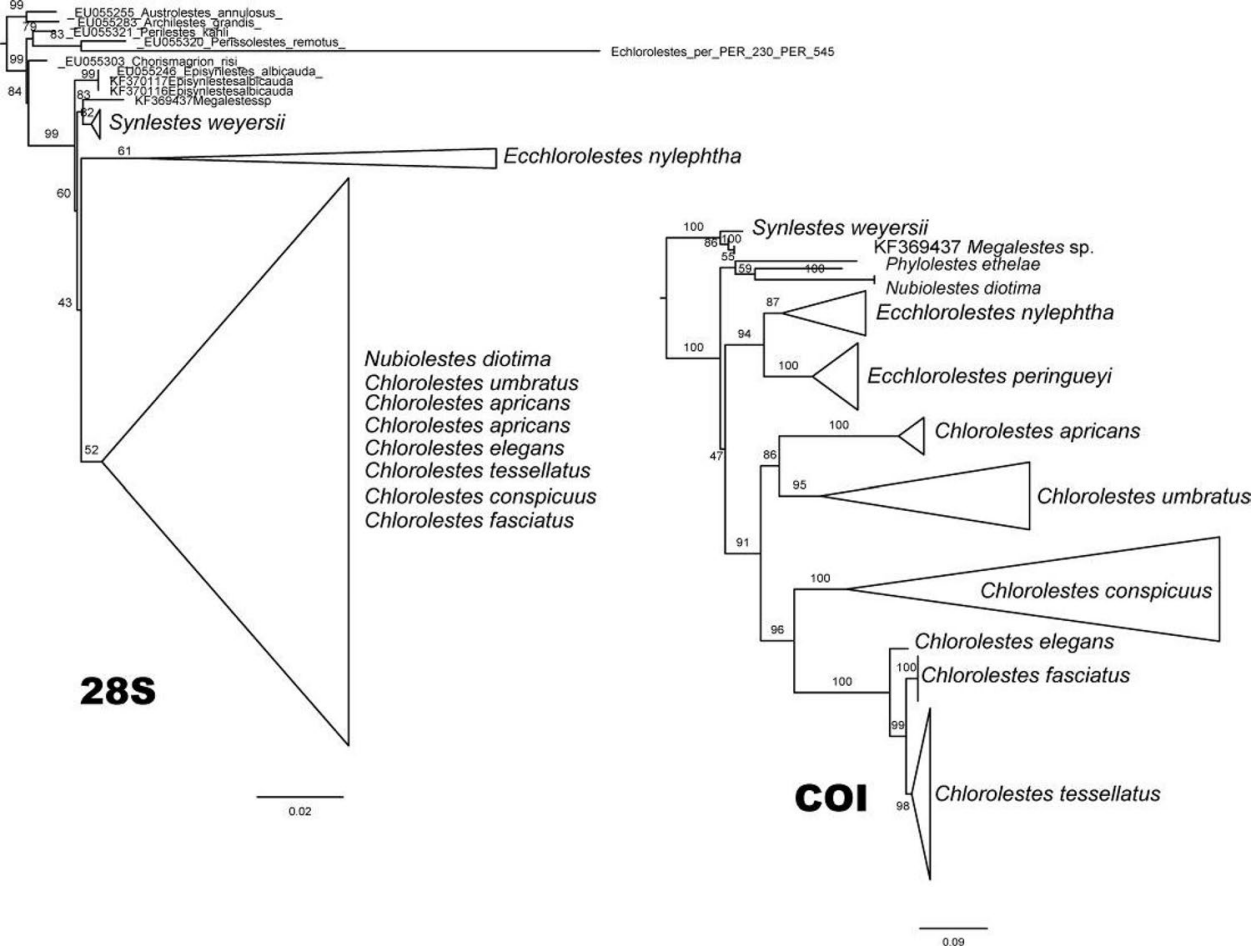
Gene trees of COI and 28S sequences based on IQtree consensus tree, with bootstrap values reported above branches.

Synlestidae *s.s.* was recovered with 77% bootstrap support. The earliest branching lineages in the topology were *Synlestes* and *Episynlestes*. *Ecchlorolestes* and *Chlorolestes* were recovered as a clade (69%), with the inclusion of *Nubiolestes, Megalestes* and *Phylolestes* (Fig. 7). The NCBI sequence of *E. nylephtha* was of 28S only (Fig. 8), which may explain why this sequence did not fall within the clade comprising the remaining members of this species. *Phylolestes* was recovered as sister to *Nubiolestes* + *Chlorolestes* (68%). Within *Chlorolestes*, *C. umbratus* was recovered as sister to the remaining *Chlorolestes* (84%). *C. apricans* was recovered as sister to (*C. conspicuus* (*C. elegans* (*C. fasciatus* + *C. tessellatus*))) (73%). The NCBI sequence for *C. tessellatus* was 28S only (Fig. 8), which may explain why it did not group with the remaining *C. fasciatus*.

The median spine located on the ligula of the male secondary genitalia seems to have evolved once, as did a tooth on the caudal appendages. The hood-like structure on the male ligula and evenly spaced concavities between ovipositor teeth were synapomorphies for the grouping of *C. elegans, C. fasciatus, C. tessellatus* (and *C. draconicus*, although not sequenced here). The presence of a basal spine on the cercus, an ovipositor with small teeth equidistant from one another, and a medial spine on the ligula, with RP_3_ originating before the subnodus were synapomorphies for both species of *Ecchlorolestes.*

**Figure 9:**
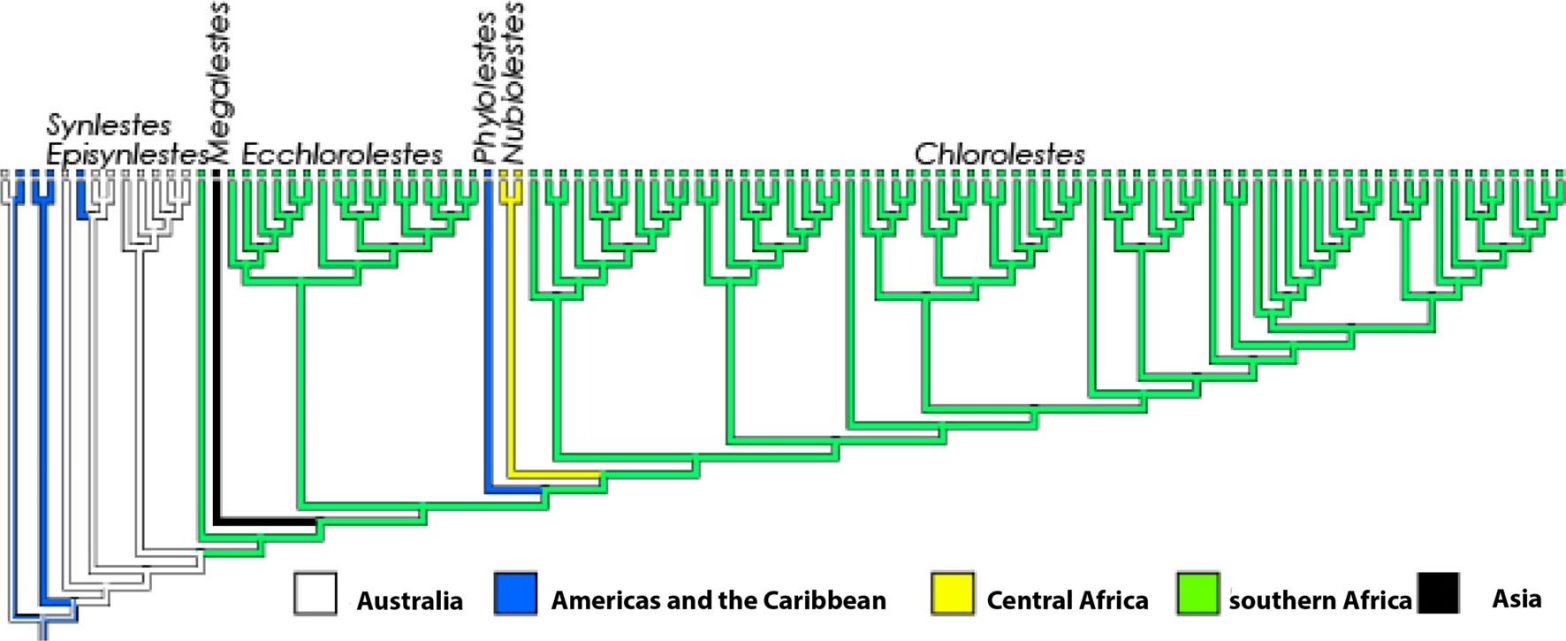
Mesquite ancestral state mapping tree suggesting biogeographical ranges for present and ancestral groups.

Biogeographical analyses using the parsimony ancestral state reconstruction function in Mesquite suggest a single African origin of Synlestidae (Fig. 9). *Megalestes,* recovered as a likely sister to the remaining Synlestidae, occurs in the Oriental Region, and our topology suggests that *Phylolestes* has an independent bioegeographical origin into the New World, most likely due to dispersal into the Caribbean.

## Discussion

### Biogeography

The present-day distributions of the Synlestidae—and Odonata in general—reflect millions of years of geographic isolation of South Africa from other areas of the continent, and high-mountain building in the south-west in particular, coupled with a lack of glaciation events for over 200 million years. This has contributed to considerable speciation and endemism^6,17^, particularly in the Cape Floristic Region (CFR), but also in the high KwaZulu-Natal Drakensberg mountains^6^. Although some individuals of Synlestidae species do occur at low elevations (e.g. *C. fasciatus* at near sea level at Mtamvuna), most representatives of the family commonly occur at higher elevations^18^, with higher elevations being common for Neotropical *Phylolestes*. Perilestidae, however, often occur at low elevations in the Amazon basin. In a warming global climate, the preference for high elevations may leave some montane specialists, such as *C. draconicus* without suitable habitat^6^. Isolated populations, such as that of *C. tessellatus* in Sevenweekspoort, at least a hundred kilometers from the nearest population, with arid and unsuitable habitat in between^17^, may face a similar isolationist situation.

*Ecchlorolestes* was first erected as a genus in 1937 by Barnard^12^. In 1962, Pinhey^19^ suggested that at least *E. nylephtha* should be considered as a member of *Chlorolestes*, but this was reversed^20^. For example, the two species in the genus are unlike *Chlorolestes* in their possession of a distinct basal tooth on the superior anal appendages^16^. The tooth is stubby in *E. peringueyi* (Fig. 4L) and narrower in *E. nylephtha* (see^16^ for figures of anal appendages, and Fig. 4K here). However, other closely related taxa also have a similar tooth to *Ecchlorolestes*, such as the Australian *Synlestes weyersii*, whose teeth on the superior appendages greatly resemble *E. peringueyi*. In the Australian lestid *Austrolestes cingulatus* (Burmeister), the teeth are similar, although each tooth is much shorter and stubbier than *E. peringueyi*. Perhaps the presence of a tooth on the superior appendage has simply been lost and gained multiple times, although this requires testing with a thorough morphological evaluation, as animal genitalia evolve rapidly with respect to other morphological traits due to sexual selection^21^. The differences in the ovipositor armature between *Chlorolestes* (robust teeth, Fig. 5C–I) and *Ecchlorolestes* (teeth small, Fig. 6) may be an adaptation to laying in soft tissue (lichens for *E. peringueyi*, and ferns/mosses for *E. nylephtha*), in comparison with harder tissue for the southern African *Chlorolestes* (terminal twiglets of sclerophyllous vegetation). A phylogenetic hypothesis based on fossil and extant zygopteran taxa would provide further insight into the evolution of synlestid morphology.

In general, Chlorolestinae have very petiolate wings, and occur in association with montane streams, especially deposition pools. Their geological history is not well known, but there are fossils of putative Synlestidae, such as *Eolestes*^22–24^ from the Eocene (roughly 56–34 million years ago) and *Gaurimacia sophiae*^25^ from the Late Jurassic, Early Cretaceous (roughly 145 mya).

### Extant Synlestidae in the new world

There is but one New World representative of Synlestidae, *Phylolestes* in the Caribbean: is this taxon there due to dispersal? The fossil *Eolestes* described by Cockerell^22^ and considered by Nel and Paicheler^23^ to be a putative Synlestidae, was recovered from the New World, in the United States of America. Perhaps Synlestidae species were once more widespread, but have since gone extinct, except for representatives in the Caribbean, Australasia, and southern Africa. If Perilestidae are indeed Synlestidae, as our topology and morphological data suggest, then the Neotropical distribution of *Phylolestes* may instead reflect an ancestral range that spanned Gondwana. The geological age of Hispaniola is Late Cretaceous to early Cenozoic^26^. Perhaps *Phylolestes* colonized or speciated on this island, while other Neotropical members of the family went extinct. An autapomorphy for Synlestidae + Perilestidae is the strongly arched CuP (Fig. 2) at its base where it meets the extremity of the quadrangle. With this revised status, New World Synlestidae are then comparatively the most species rich with 21 species compared with taxa from Asia (19), Africa (9) and Australia (7).

Do larval characteristics support or refute uniting Perilestidae and Synlestidae? We considered all larval descriptions of perilestids^27–30^ and synlestids known to us^31–36^. The caudal lamellae in all genera are very similar in all genera, but the larva of *Megalestes* (Synlestidae) differs more from all of the other genera (*Perilestes*, *Perissolestes*, *Phylolestes*, *Chorismagrion*, *Episynlestes*, *Synlestes* and *Nubiolestes*) referenced above. The shape of the labium and delicate spider-like legs in *Megalestes* differ from all of the other genera listed above. In summary, the differences among genera within Synlestidae, based on the published literature, seem to be at least as great, or greater, than differences between Perilestidae and Synlestidae. Any argument to sustain Perilestidae as separate from Synlestidae based on larvae does not seem to be particularly significant. Arguments as to differing altitude preferences also do not seem to be valid reasons for maintaining Perilestidae as separate from Synlestidae, as these can vary even with a single genus. For example, C. draconicus only occurs at high elevations, but many others either occur in a wide elevational range, (*C. fasciatus*) or even at low elevations (*C. tessellatus, C. conspicuus*). It is therefore unlikely that elevation provides a valid reason for maintaining the two families as separate.

### Taxonomy and the status of the southern African genera in Synlestidae

Based on our results, *Ecchlorolestes* and *Chlorolestes* are valid taxonomic groups, as also suggested by larval morphology^34^. Some subgroups within *Chlorolestes* have been suggested based on wing banding. *Chlorolestes umbratus, C. tessellatus, C. fasciatus, C. elegans* possess banded wings in all or some populations. Our topology suggests that this banding is perhaps not so much a reflection of evolutionary history as localized selection pressure. For example, wing banding in *C. tessellatus* populations varies according to geographical area, with some populations in the Eastern Cape with heavily banded wings, and others, for example in KwaZulu-Natal with no banding at all. One population, also in KwaZulu-Natal, has very weakly banded wings^37^. Similar polymorphism in wing maculation occurs in *Sinolestes editus* Needham and some species of *Orolestes* McLachlan, 1895, which belong to the family Lestidae)^38,39^. Here, we propose sinking the family Perilestidae within the Synlestidae based on (a) their position as sister to the large clade containing *Nubiolestes* and the Synlestidae, (b) morphological characters, such as the strongly arched CuP at its base where it meets the extremity of the quadrangle and ovipositor, described above.

## Conclusions

*Ecchlorolestes* and *Chlorolestes* are monophyletic. We suggest that given the molecular topology and morphological data, Perilestidae should be considered members of the Synlestidae. *Phylolestes* is sister to *Chlorolestes*, suggesting that there was an African origin of the clade containing *Nubiolestes, Ecchlorolestes, Phylolestes* and *Chlorolestes*, and subsequent dispersal to the Caribbean by *Phylolestes,* unless Perilestidae are also included within the family, in which case perhaps a broader origin is possible, as supported by the fossil evidence and Mesquite analysis.

## Supplementary information

Figure S1. Living specimens of Synlestidae. Male imago of: (A) Chlorolestes apricans, (B) C. conspicuous, (C) C. draconicus, (D) C. elegans, (E) C. fasciatus, (F) C. tessellatus, (G) C. umbratus, (H) Ecchlorolestes nyleptha, and (I) E. peringueyi. Photographs by JPS (B, E, F, G, H, I) and MJS (A, C, D)

Figure S2. Maps showing the South African distributions of: (A) Chlorolestes (crosses), and Ecchlorolestes (grey-filled triangles species in South Africa, (B) C. apricans (crosses), C. draconicus (dots), C. elegans (squares) and C. umbratus (triangles), (C) C. conspicuus (dots), (D) C. fasciatus (dots), C. tessellatus (dots) and (E) Ecchlorolestes nylephtha (squares) and E. peringueyi (triangles).

Table S1. Specimen selection for present study.

Table S2. National Center for Biotechnology Information (NCBI) GenAccession numbers of species added to the 28S and COI sequences in our specimen dataset.
