# Supplementary figures and images for: Phylogeny of the Synlestidae (Odonata: Zygoptera), with an emphasis on Chlorolestes Selys and Ecchlorolestes Barnard
Source: Sci Rep. 2020 Sep 15;10:15088. doi: 10.1038/s41598-020-72001-x (PMC7492436; doi:10.1038/s41598-020-72001-x)

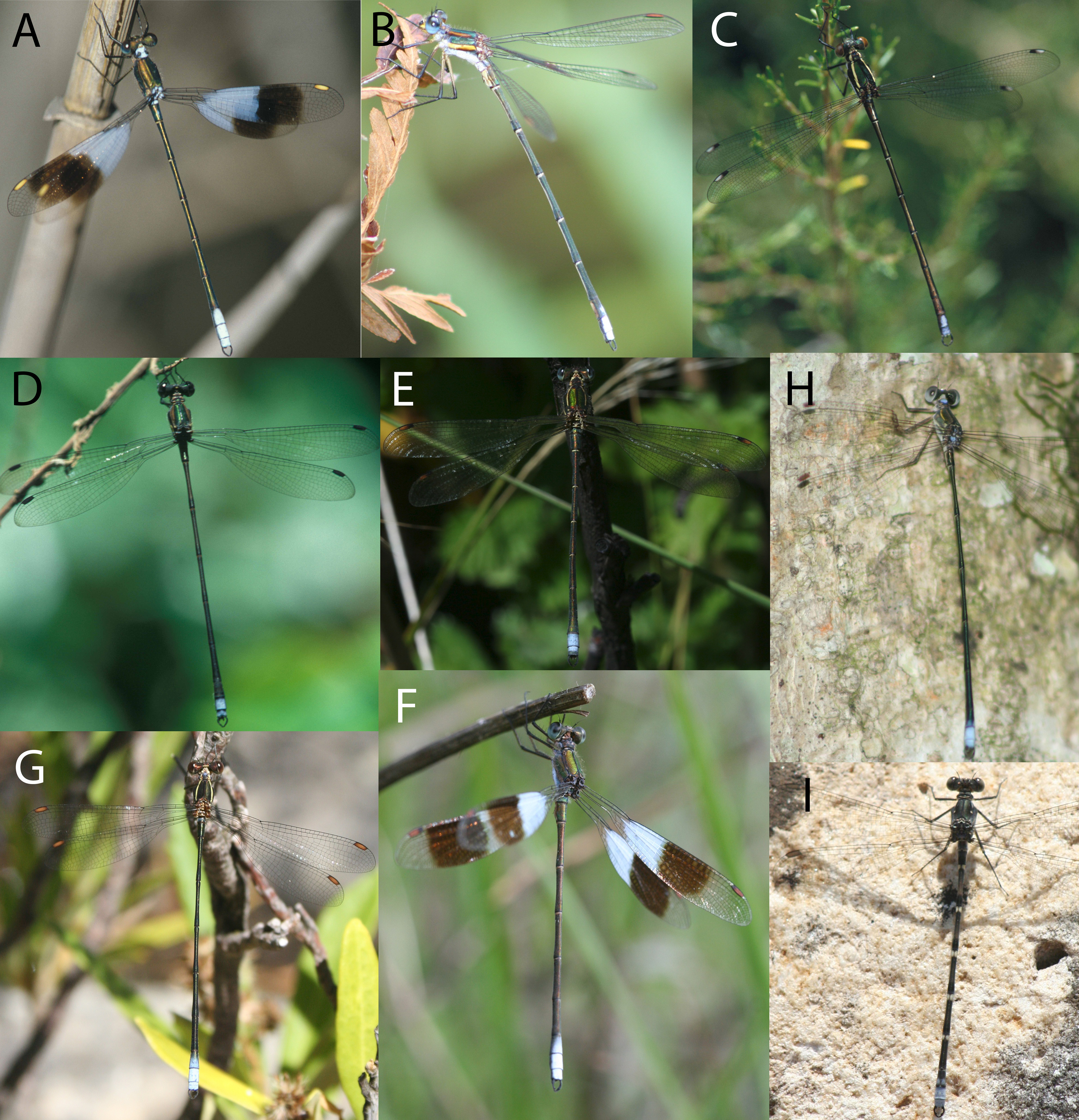

Supplement: Supplementary file 1 — Figure S1. Living specimens of Synlestidae. Male imago of: (A) Chlorolestes apricans, (B) C. conspicuous, (C) C. draconicus, (D) C. elegans, (E) C. fasciatus, (F) C. tessellatus, (G) C. umbratus, (H) Ecchlorolestes nyleptha, and (I) E. peringueyi. Photographs by JPS (B, E, F, G, H, I) and MJS (A, C, D) [file 41598_2020_72001_MOESM1_ESM.jpg]

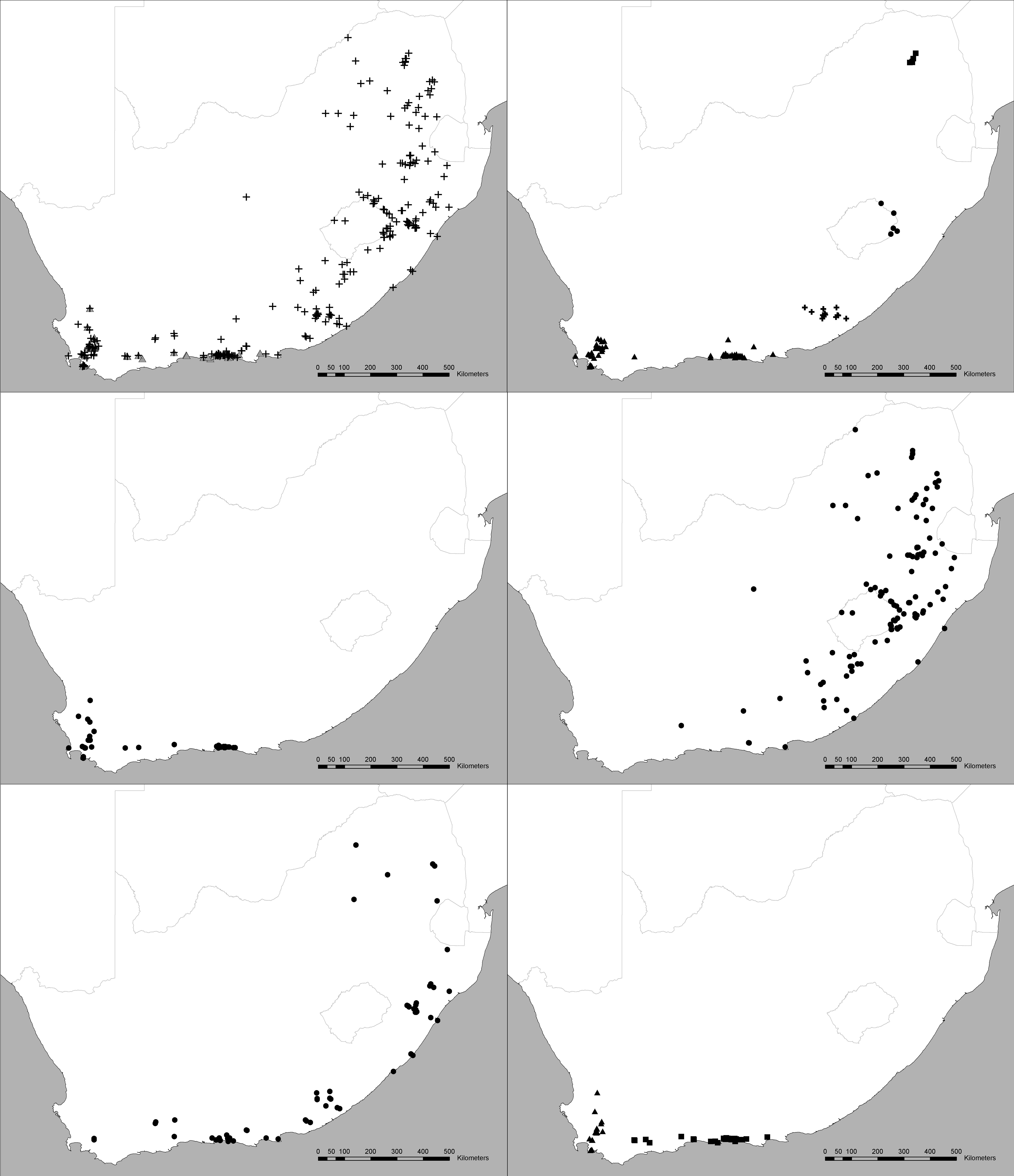

Supplement: Supplementary file 2 — Figure S2. Maps showing the South African distributions of: (A) Chlorolestes (crosses), and Ecchlorolestes (grey-filled triangles species in South Africa, (B) C. apricans (crosses), C. draconicus (dots), C. elegans (squares) and C. umbratus (triangles), (C) C. conspicuus (dots), (D) C. fasciatus (dots), C. tessellatus (dots) and (E) Ecchlorolestes nylephtha (squares) and E. peringueyi (triangles). [file 41598_2020_72001_MOESM2_ESM.jpg]
